# New Estimators and Guidelines for Better Use of Fetal Heart Rate Estimators with Doppler Ultrasound Devices

**DOI:** 10.1155/2014/784862

**Published:** 2014-01-29

**Authors:** Iulian Voicu, Sébastien Ménigot, Denis Kouamé, Jean-Marc Girault

**Affiliations:** ^1^Signal & Imaging Group, University François Rabelais of Tours, PRES Loire Valley University, UMR INSERM U930, 7 Avenue Marcel Dassault, 37200 Tours Cedex, France; ^2^Inserm, U930, 10 Boulevard Tonnellé, BP 3223, 37032 Tours Cedex, France; ^3^IRIT UMR 5505, Université Paul Sabatier Toulouse 3, 118 Route de Narbonne, 31062 Toulouse Cedex 9, France

## Abstract

Characterizing fetal wellbeing with a Doppler ultrasound device requires computation of a score based on fetal parameters. In order to analyze the parameters derived from the fetal heart rate correctly, an accuracy of 0.25 beats per minute is needed. Simultaneously with the lowest false negative rate and the highest sensitivity, we investigated whether various Doppler techniques ensure this accuracy. We found that the accuracy was ensured if directional Doppler signals and autocorrelation estimation were used. Our best estimator provided sensitivity of 95.5%, corresponding to an improvement of 14% compared to the standard estimator.

## 1. Introduction

Continuous monitoring of fetal parameters has shown their advantages in estimating fetal wellbeing [1]. According to the report of the Society for Maternal-Fetal Medicine [2], continuous fetal heart rate monitoring has reduced infant mortality. The development or the improvement of noninvasive methods dedicated to continuous fetal monitoring is therefore of major interest.

One important parameter in assessing fetal wellbeing is the variability of the fetal heart rate. This parameter, which corresponds to the variation between intervals of two consecutive heart beats, is an indicator of central nervous system development [3–5]. It characterizes fetal behavior states [6–8] and can be an indicator of further neurological evolution [9]. Variability analysis provides a good indication of fetal distress [10] and identifies fetuses with intrauterine growth retardation [11]. According to Dawes criteria [12], variability of 4 ms is a predictor of the lack of acidosis, while a value of 2.6 ms is critical for the fetus. In the normal fetal heart rate range (110–160 bpm), a time variability of 4 ms corresponds to a cardiac frequency variability of 0.81 bpm, while a time variability of 2.6 ms corresponds to a cardiac frequency variability of 0.53 bpm (the values of 0.53 bpm and 0.81 bpm are obtained as follows: 60/(60/110 − 2.6 ms) − 110 = 0.56 bpm and 60/(60/110 − 4.0 ms) − 110 = 0.81 bpm, resp.). Other authors [13] suggest that it is necessary to estimate the heart rate with an accuracy of 0.25 bpm in order to analyze fetal heart rate variability correctly. Reliable estimation of fetal heart rate and hence of heart rate variability is therefore essential.

Several methods are available to assess the fetal heart rate. These methods differ both at the Doppler signal level (directional or nondirectional) and at the level of the algorithm that estimates the heart rate. For example, existing devices on the market such as Sonicaid Oxford (Oxford Sonicaid Instruments, Abington, UK) [14], Hewlett-Packard 8030A (Hewlett-Packard, Palo Alto, CA, USA) [15], and Philips Avalon F40 (Philips, Amsterdam, Netherlands) use the envelope of the nondirectional Doppler signal. Other authors [16, 17] have used the envelope of the directional Doppler signal. Several algorithms based on autocorrelation are commonly used to estimate the heart rate (Oxford Sonicaid, Hewlett-Packard 8030A, and Philips Avalon F40). These algorithms have been applied either directly to the Doppler signal envelope (directional or nondirectional) or to the signals resulting from discrete wavelet decomposition of the envelope. In the latter case, the final estimated heart rate involves the combination of different estimates [16].

In this study, we first verified whether the pulsed Doppler techniques currently used in commercial devices ensure such an accuracy of 0.25 bpm, and we propose here some recommendations regarding parameter settings. We also compared the techniques used in commercial devices with other pulsed Doppler techniques that use directional Doppler signals. We evaluated the efficacy of all these techniques empirically (error of estimation of the fetal heart rate, sensitivity, and false negative rate).

The originality of this study lies in the recommendations on the parameter settings of the system and in the description of the individual limitations of each technique. Finally, to improve detection probability, a new method based on the combination of heart rates obtained from directional signals is proposed.

## 2. Materials and Methods

In this section, we describe the Doppler system we developed, the patients, and the synthetic signals used for the comparison of each estimator. The synthetic signals were inferred from real signals. Finally, we present the various existing techniques for estimation of fetal heart rate and we present a new technique based on a combined procedure.

### 2.1. The Doppler System

In order to evaluate fetal wellbeing objectively and to classify the fetus, we codeveloped the pulsed, multitransducer, multichannel Doppler Actifoetus unit with Althaïs Technologies (Tours, France).

Our system comprised a personal computer (PC) and our Actifoetus unit. The Actifoetus unit contained three groups of four transducers and a Doppler acquisition board. The detailed operating functions of the acquisition board were presented in [18].

The transducers exploring the fetal heart were non-focused and monoelement. They were circular in shape, with a diameter of 13.5 mm and an acoustic power of 1 mW/cm^2^. Geometrically, the transducers were located at the center of gravity and at the top of an equilateral triangle with sides measuring 40.7 mm.

The transducers were placed on the mother's abdomen. They transmitted a sinusoidal pulse at 2.25 MHz with a pulse repetition frequency (PRF) of 1 kHz. Note that a theoretical accuracy of 60/2/1000 = 0.03 bpm can be achieved with this value of 1 kHz and accuracy can be still further improved by performing interpolation of the correlation function. The wave was propagated through the mother's abdomen towards the fetal heart. The backscattered signal was recorded from five different depths, annotated *D*_1_,…, *D*_5_. Note that only one channel was considered in the present study.

The ultrasound signal received was converted into an electrical signal and amplified to compensate for the attenuation of 1 dB/cm/MHz. The signal was then demodulated in phase (*I*) and quadrature (*Q*) [19]. After demodulation, the signals were digitized. The digital outputs of the converters represented the digital Doppler signal.

### 2.2. Patients

The Doppler signals were acquired at the CHRU “Bretonneau” Tours, France. The consent of each patient was obtained and the study was approved by the Ethics Committee of the Clinical Investigation Centre for Innovative Technology of Tours (CIC-IT 806 CHRU of Tours). Patients were older than eighteen years and all pregnancies were single. The recordings were made during the twenty-fifth and fortieth gestational weeks. Evolution during pregnancy was normal for all fetuses.

### 2.3. Simulation

**Figure 1:**
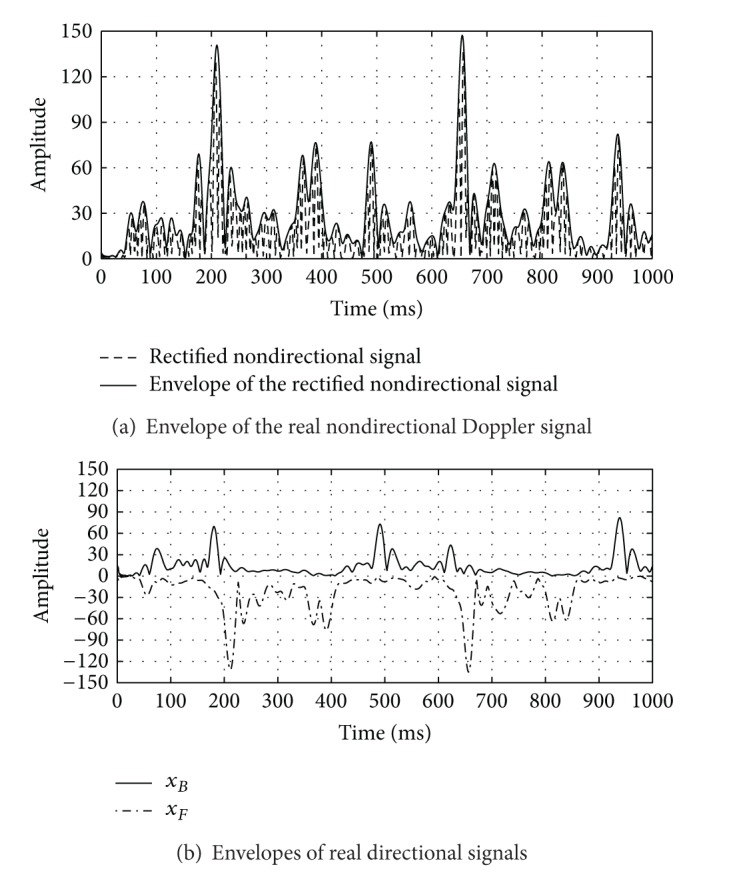
A real Doppler signal (PRF = 1 kHz) of 1000 ms, recorded with the second transducer in the fourth-channel: (a) Doppler signal (dashed line) and its envelope (solid line); (b) the envelopes of directional signals corresponding to ultrasound scatters approaching to the transducer (solid line) and moving away from the transducer (dash-dot line), respectively.

Because it was difficult to quantify the effectiveness of the estimation techniques directly on real signals and because there was no suitable model, we generated synthetic signals. These synthetic signals were used as a ground-truth to evaluate the effectiveness of each estimator. To make these signals as realistic as possible, we proceeded in two stages: an analyzing stage deducing the characteristics of the real Doppler signal envelope and a synthetic phase providing realistic simulated signals. Figures 1(a) and 1(b) show 1000 ms of the envelope of a real nondirectional Doppler signal and the two envelopes of corresponding directional signals obtained from the *I* and *Q* signals [19, 20]. The synthetic envelope signal was calculated as follows:

(1) $$\begin{array}{c} x_{e}\left(t\right)=x_{B}\left(t\right)+x_{F}\left(t\right), \end{array}$$

where *x*_*B*_(*t*) and *x*_*F*_(*t*) are the envelopes of directional Doppler signals produced by the scatters that approach and move away from the transducer, respectively.

We verified (Figures 1(a) and 1(b)) that the envelope of the real nondirectional Doppler signal had the signatures of both envelopes of the directional signals. For example, at around 500 ms, the envelope of the nondirectional signal was mainly influenced by scatters that approached the transducer, while at around 400 ms, we observed the influence of movements away from the transducer. The alternating influence of these two movements determined the envelope of the nondirectional signal.

#### 2.3.1. Analysis of Real Directional Signals

**Figure 2:**
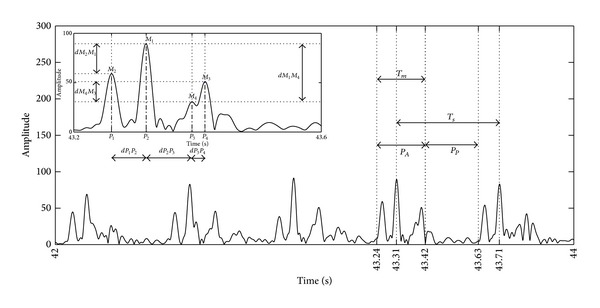
Envelope of a real directional Doppler signal of 2000 ms. The parameters defining the synthetic signal are the amplitudes and durations of peaks, the lag, and the differences in amplitude of two consecutive peaks over time.

Figure 2 shows 2000 ms of the envelope of a real directional Doppler signal. In order to find the important parameters required for the synthesis of this signal, we extracted its intrinsic features (the number and amplitudes of the peaks, the lags between the peaks, and the differences in amplitude between the peaks). The values of these parameters were evaluated by considering a quasiconstant fetal heart rate.

Figure 2 represents a sequence of several patterns. These patterns were made up of peaks that corresponded to cardiac wall and valve movements of the fetal heart. As suggested by Shakespeare et al. [21], although six peaks (atrial contraction, ventricular contraction, opening and closing of the mitral valves, and opening and closing of the aortic valves) could be detected theoretically, only a few peaks were in practice detected in the nondirectional Doppler signal. From our analysis, it appeared that the most likely pattern was that with four peaks. Note that this signature composed of four peaks could vary considerably from one beat to another, and it was similar to that identified by Jezewski et al. [13]. Among all these patterns, the most likely was the pattern with peaks in the order 2143; that is, the highest peak *M*_1_ was in second position, the second highest peak *M*_2_ was in first position, and so forth. For the 2143-pattern, we evaluated the amplitudes of each peak (*M*_1_, *M*_2_, *M*_3_, and *M*_4_), the peak durations (*T*_1_, *T*_2_, *T*_3_, and *T*_4_), the lags between two consecutive peaks (*dP*_1_ *P*_2_, *dP*_2_ *P*_3_, and *dP*_3_ *P*_4_), and the differences in amplitude between two consecutive peaks (*dM*_2_ *M*_1_, *dM*_1_ *M*_4_, and *dM*_4_ *M*_3_). The results of this statistical analysis are reported in Table 1.

As the patterns observed in Figure 2 were noisy, we decided to assess the noise level in order to simulate noisy synthetic Doppler signals. We assessed the signal to noise ratio (SNR) as follows:

(2) $$\begin{array}{c} \text{SNR}=10\cdot \log_{10}\left(\frac{P_{A}}{P_{P}}\right), \end{array}$$

where *P*_*A*_ and *P*_*P*_ are the powers of the active and passive regions, respectively. We considered the active region as the area containing the pattern peaks, whereas there were none in the passive region. Using (2), we found that the SNR calculated on our real signals corresponded to a Gaussian law: $\text{SNR}\sim N(11,{\left(\sqrt{2.5}\right)}^{2}(\text{dB}))$.

#### 2.3.2. Synthesis of Synthetic Directional Signals

Analysis of the envelope of directional signals showed the presence of four peaks, which appeared in order 2143 inside the periodic patterns. The synthesis of such a signal must account for these characteristics. Equation (3) shows the two possible components of such a signal:

(3) xB(t)={b(t)+∑i=14Misin(2πfi(θ+Ti2))RectTi(θ),                 ∀t∈[0,Tm],b(t),             ∀t∈(Tm,Ts],

where *b*(*t*) is the noise, *M*_*i*_ is the peak amplitude, *f*_*i*_ = 1/(2*T*_*i*_) is the peak frequency, Rect_*T*_*i*__(*θ*) is the unit rectangular function centered on *T*_*c*_*i*__ with width *T*_*i*_ and *θ* = *t* − *T*_*c*_*i*__, *T*_*m*_ is the pattern duration, and *T*_*s*_ is the synthetic signal period. We set a constant interval *T*_*s*_ between the highest peaks of two consecutive patterns of the synthetic signal, as illustrated in Figure 2. We also chose the *T*_*m*_ pattern period as 50% of the synthetic cardiac cycle period *T*_*s*_, since this period can vary between 40 and 60% [22].

Using (3), we generated two synthetic noisy envelopes corresponding to the envelopes of the directional signals. The envelope of the nondirectional synthetic signal was modeled using (1), being the sum of the envelopes of the both directional synthetic signals. In order to simplify our study, only *x*_*B*_(*t*) was calculated, *x*_*F*_(*t*) being a delayed and amplified version of *x*_*B*_(*t*). To simulate realistic signals, we introduced a lag *τ* between the directional components:

(4) $$\begin{array}{c} x_{F}\left(t\right)=\alpha x_{B}\left(t-\tau \right), \end{array}$$

where *x*_*F*_(*t*) and *x*_*B*_(*t*) are the envelopes of directional signals and *τ* is the lag between the two envelopes. *α* is a factor that represents the amplitude ratio between the two types of envelope. From Figure 1, *τ* ≈ 40 ms and *α* ≈ 2.

### 2.4. Estimators

**Figure 3:**
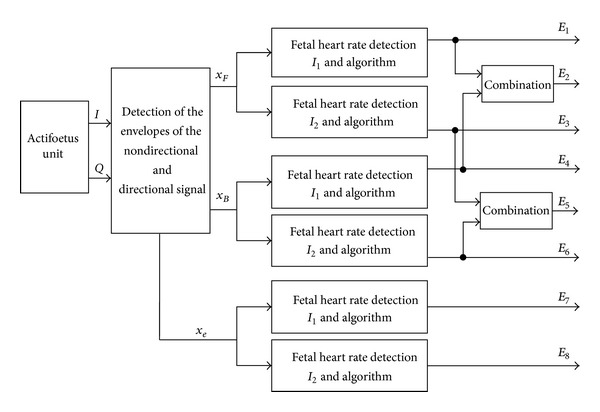
General diagram of the Doppler data processing acquired using one transducer and one channel. *x*_*F*_ and *x*_*B*_ represent the envelopes of the directional signals, and *x*_*e*_ represents the envelope of the nondirectional signal. *I*_1_, *I*_2_ are the two autocorrelation estimators.

In this section, we describe the different estimators used in our study. Each estimator that was based on the autocorrelation function was denoted by *E*_*i*_, *i* = 1,…, 8, as illustrated in Figure 3. Each estimator operated on different signals: *x*_*F*_(*t*), *x*_*B*_(*t*), and *x*_*e*_(*t*).

Devices existing on the market currently use the *x*_*e*_(*t*) envelope and autocorrelation. The estimators that used these configurations were *E*_7_ and *E*_8_. The mathematical expression of the two autocorrelation estimators is given in [23] and is represented thereafter by

(5) $$\begin{array}{c} I_{1}\left(t,k\right)=\frac{1}{W}\sum_{n=0}^{W-|k|-1}x\left(t,n\right)\cdot x\left(t,n+k\right);\\ I_{2}\left(t,k\right)=\frac{1}{W}\sum_{n=0}^{W-1}x\left(t,n\right)\cdot x\left(t,n+k\right), \end{array}$$

where *W* is the size of the analyzing window, *t* is the time for which the estimator is computed, and *k* is the lag. *x*(*t*) represents one of the signals analyzed (*x*_*F*_(*t*), *x*_*B*_(*t*), or *x*_*e*_(*t*)).

We tested other estimators (*E*_1_,…, *E*_6_) which used directional signals *x*_*F*_(*t*) and *x*_*B*_(*t*), together with *I*_1_ and *I*_2_.

#### 2.4.1. Algorithm

The algorithm to estimate the fetal heart rate was the same for all three signals (*x*_*e*_(*t*), *x*_*F*_(*t*), and *x*_*B*_(*t*)). The steps of the algorithm were as follows.

(1) Extract from each signal under consideration (*x*_*e*_(*t*), *x*_*F*_(*t*), or *x*_*B*_(*t*)) a limited number *W* of samples, *W* being the window size.
(2) Compute *I*_1_(*W*) and *I*_2_(*W*).
(3) Using an empirical threshold, detect the position of *N* peaks in *I*_1_(*W*) and *I*_2_(*W*).
(4) From the position of *N* peaks of *I*_1_(*W*) and *I*_2_(*W*), determine the durations *D*_*i*_ between consecutive peaks with *i* = 1,2,…, *N* − 1.
(5) Calculate the *N* − 1 cardiac frequencies with CF_*i*_ = 60/*D*_*i*_, *i* = 1,2,…, *N* − 1. This conditional test limits the number of cardiac frequencies CF_*i*_ estimated from *I*_1_ or *I*_2_ in the average computation. This conditional test also permits removal of cardiac frequency estimates that are half the expected value, as are sometimes observed (Shakespeare et al. [21]).
(6) Calculate the average cardiac frequency (FHR) from CF_*i*_ not exceeding 35 bpm [13]:
  (6) $$\begin{array}{c} \text{FHR}=\frac{1}{N-1}\sum_{i}^{N-1}\text{C}{\text{F}}_{i}. \end{array}$$

As an illustration, consider a window of 4.096 s. Whenever the cardiac frequency was 240 bpm, 16 peaks were observed in the autocorrelation function. Using an empirically set threshold, the duration between each peak was measured (*D*_1_ = 0.250 s,…, *D*_15_ = 0.250 s) and cardiac frequencies of CF_1_ = 60/0.250 bpm,…, CF_15_ = 60/0.250 bpm were estimated. The average cardiac frequency was obtained by FHR = (1/15)∑_*i*_^15^CF_*i*_ = 240 bpm. Note that 4 peaks were observed with 60 bpm and *D*_1_ = 60/1.0 = 1.0 s,…, *D*_3_ = 60/1.0 = 1.0 s were estimated. The average cardiac frequency was obtained by FHR = (1/3)∑_*i*_^3^CF_*i*_ = 60 bpm.

Thus the proposed algorithm correctly worked in the range of 60–240 bpm. However, the standard deviation of the FHR estimation was not the same for its extreme values since in one case the average was obtained with three values whereas the average was obtained with fifteen values in the other.

Note that such an algorithm is not perfect since it is hypothesised that the FHR is constant during the process. Sometimes the second peak of the autocorrelation can be lower than the third and the FHR estimate is incorrect. A process must be performed to remove outliers.

#### 2.4.2. Elimination of Outliers

In order to eliminate outlier estimates associated with estimator dysfunction, we introduced a postprocessing step. This postprocessing step was applied only in the case of real signals. An estimate was considered to be an outlier if it laid outside the statistic computed from 40 previous estimates, or if it differed between two consecutive analysis windows by 35 bpm.

#### 2.4.3. Combination

In order to improve the effectiveness of the FHR estimation, we combined estimations. For *E*_2_ and *E*_5_, the two values of the fetal heart rate estimated on signals *x*_*F*_(*t*) and *x*_*B*_(*t*) were combined. The estimate on the two signals was achieved using *I*_1_ or *I*_2_. The combination rule we used was as follows:

- if the heart rate was detected on a single signal, the combined value took this value;
- if the heart rate was detected on both signals, the combined value was the average of the two values.

Note that, in contrast to Kret's study [16], we combined the fetal heart rates estimated on both directional Doppler signals, while Kret's technique was based on combination of fetal heart rate estimations computed after discrete wavelet decomposition of the envelope of the directional Doppler signal. Since Kret's technique was applied only for continuous Doppler signals, it was not taken into consideration in our study.

## 3. Results

To find the best estimators and the conditions in which they could be used, we performed a series of simulations and experiments. Using simulations, we sought configurations that ensured an error of estimation, that is, the expected accuracy below 0.25 bpm, the highest sensitivity, and the lowest false negative rate. Experimentally, we sought the best configuration for optimal use of the estimator.

### 3.1. Simulated Signals

We present the results from two types of simulation. In the first series of simulations, we sought parameter settings that ensured the desired accuracy of 0.25 bpm. In the second series of simulations, we evaluated the effectiveness of each estimator in terms of true positive rate and false negative rate.

#### 3.1.1. Optimal Parameter Settings

**Figure 4:**
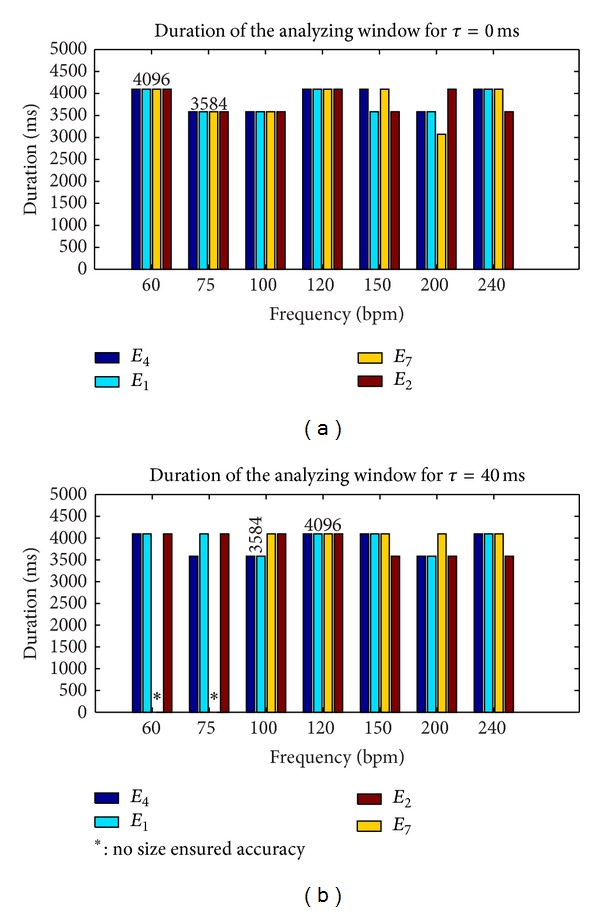
Duration of the analyzing window *W* required to reach an error of at least 0.25 bpm with the autocorrelation estimator (*I*_1_) and a SNR > 0.6 dB.

**Figure 5:**
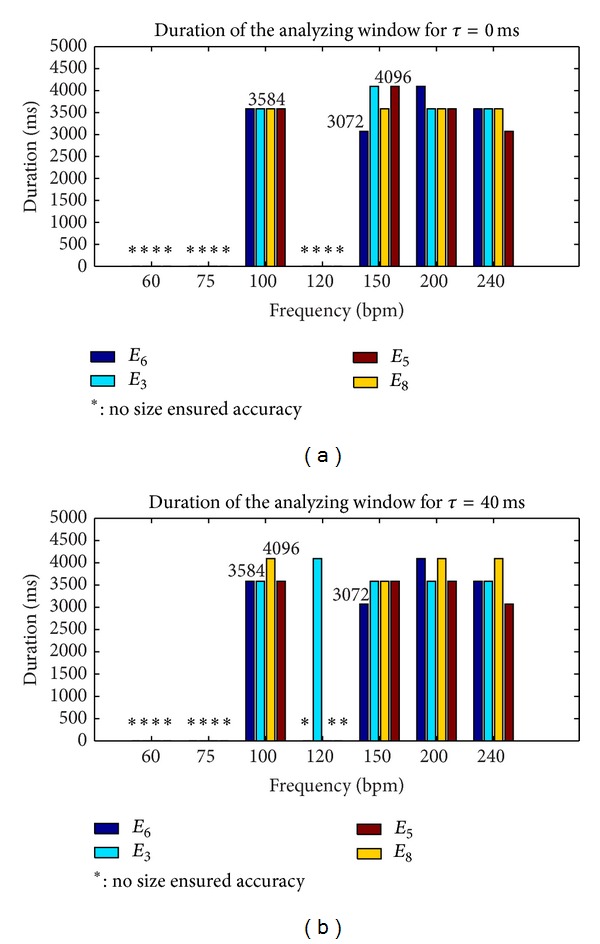
Duration of the analyzing window *W* required to reach an error of at least 0.25 bpm with the autocorrelation estimator (*I*_2_) and with a SNR > 0.6 dB.

The results presented in Figures 4 and 5 were obtained for synthetic signals of 30 s. The parameters that varied in our analysis were the periodicity of the signal *T*_*s*_, the SNR, the window size *W*, and the lag *τ*. We varied the signal periodicity *T*_*s*_ between 1000 and 250 ms, as these values corresponded to the standard range of exploration (60–240 bpm) of different fetal monitors. The SNR range varied between 0 and 14 dB, in order to include our measured SNR values on the real signals and in order to take into account the worst cases. The range of *W* size varied between 512 and 4096 ms. The highest fetal heart rate could be obtained with a window size of 512 ms, although we limited the maximum window to 4096 ms to reduce computation time.

Figures 4 and 5 show the smallest window size analyzed (*W* = 4096 ms) of all estimators tested that ensured the expected accuracy of 0.25 bpm in the range of 60–240 bpm and that ensured a SNR at least greater than 0.6 dB. Note that for estimator *I*_2_ reported in Figure 5, we showed that there was no size which ensured the desired accuracy, whatever the SNR or the frequency. To test estimator robustness in relation to the increasing complexity of the simulated signals, the lag *τ* varied between 0 and 40 ms, this value of 40 ms being taken from Figure 1.

The results derived from Figure 4 showed that accuracy for the envelope signal (estimator *E*_7_) was no longer achieved for certain frequencies, but it still was for directional signals. Finally, Figure 5, shows the best estimators (*E*_1_, *E*_2_, *E*_4_) and their respective best parameter settings (*W* = 4096) that ensured an accuracy of 0.25 bpm with a SNR > 0.6 dB in the 60–240 bpm range.

To summarize, these first results showed the superiority of *I*_1_ compared to *I*_2_ and the superiority of the envelope of directional signals compared to that of nondirectional signals. We therefore recommend the use of *I*_1_ and the estimators (*E*_1_, *E*_2_, and *E*_4_) based on the envelope of directional signals.

#### 3.1.2. Performance Levels of Estimators

In this study, the performance levels of estimators we wanted to compute were sensitivity and the false negative rate. Fetal heart rates were evaluated every 250 ms from noisy signals with *W* = 4096 ms. Sensitivity was computed with the equation: *S* = TP/(TP + FN), where TP was the true positive rate and FN was the false negative rate. Estimates of simulated heart rate were considered to be false negative if they did not ensure the expected accuracy; otherwise, they were true positive. Sensitivity and the false positive rate were evaluated as the average of 30 values. Each value was determined after analysis of a noisy signal of 30 s where sensitivity and the false positive rate had converged to the highest value and to the lowest value, respectively. Convergence was reached for a minimum SNR of 6 dB.

**Figure 6:**
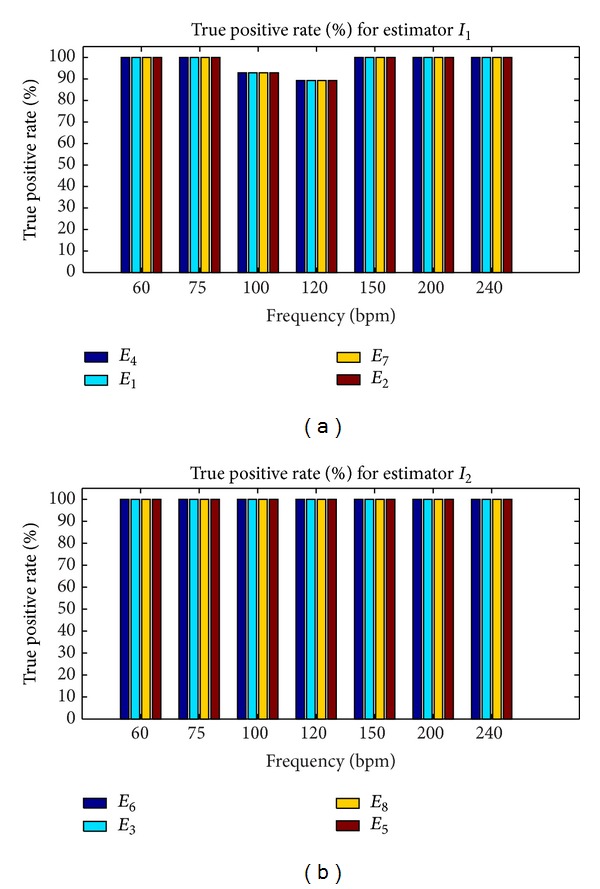
True positive rates for *I*_1_ and *I*_2_ with SNR > 0.6 dB, *W* = 4096, and an error of estimation of 0.25 bpm. (a) True positive rate for *I*_1_. (b) True positive rate for *I*_2_.

**Figure 7:**
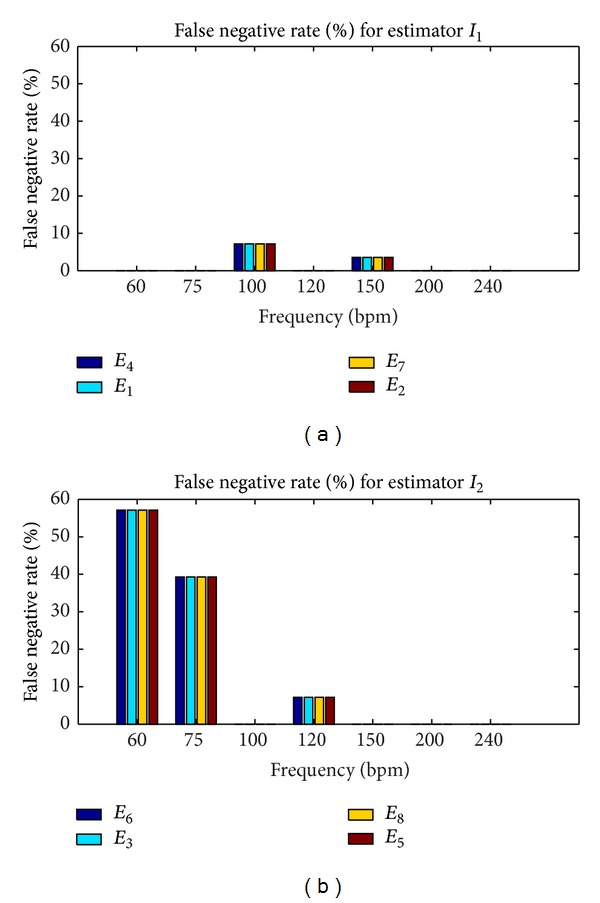
False negative rates for *I*_1_ and *I*_2_ with SNR > 0.6 dB, *W* = 4096. Accuracy of 25 bpm was obtained only for *I*_1_ whatever the frequency, whereas for *I*_2_ accuracy was obtained for only 100, 150, 200, 220, and 240 bpm. (a) False negative rate for *I*_1_. (b) False negative rate for *I*_2_.

The results of this second series of simulations are presented in Figures 6 and 7 for *W* = 4096 ms and SNR ≥ 6 dB. Note that the estimation of error of 25 bpm was obtained only for *I*_1_ whatever the frequency, whereas accuracy for *I*_2_ was obtained only for 100, 150, 200, 220, and 240 bpm.

The results set out in Figure 7 show that estimators based on *I*_1_ (*E*_1_, *E*_2_, *E*_4_, and *E*_7_) had an average (average obtained from the cardiac frequency) false negative rate of 1.5%, while those based on *I*_2_ (*E*_3_, *E*_5_, *E*_6_, and *E*_8_) presented a higher average false negative rate of approximately 14.8%. The 97.5% average true positive rate of *I*_1_ was slightly lower than that of estimators based on *I*_2_, which was 100%. Finally, when the accuracy of 0.25 bpm was reached, we observed that the estimators based on *I*_1_ were generally more accurate than those based on *I*_2_, although the average false negative rate was not zero.

**Figure 8:**
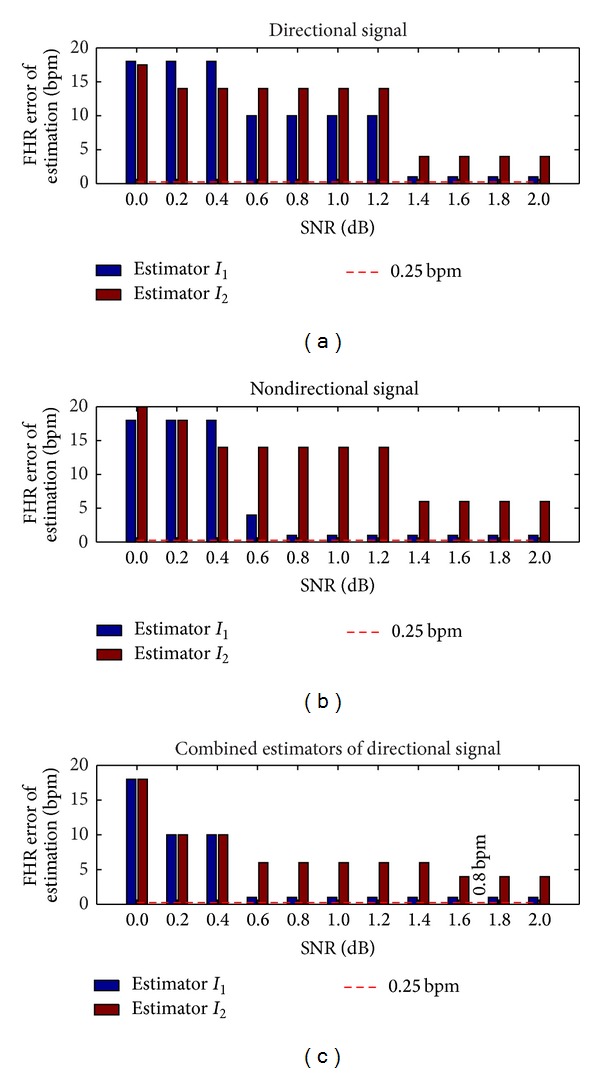
FHR error of estimation (bpm) when the false negative rate was zero, when the SNR ∈ (0 dB, 2 dB), and when the signals tested were *x*_*F*_, *x*_*B*_, and *x*_*e*_.

Figure 8 shows the error of estimation corresponding to different values of SNR when a zero false negative rate was imposed. This zero false negative rate was obtained by modifying the detection threshold, and a direct consequence was an increase in the estimation. The results derived from Figure 8 showed that the zero false negative rate for *I*_1_ was ensured for a SNR ≥ 2 dB (below the SNR measured on real signals) and for an error of estimation of 0.8 bpm. In the case of *I*_2_ and directional signals, we obtained an error of estimation of 4 bpm, whereas for a nondirectional signal it was 6 bpm.

Table 2 summarizes the effectiveness of each estimator in terms of FHR error of estimation, SNR, and average false negative rate. To reach an error of estimation, that is, the expected accuracy of 0.25 bpm, we recommend *I*_1_, that is, autocorrelation-based estimators (*E*_1_, *E*_2_, *E*_4_, and *E*_7_), the price to be paid being an average false negative rate of 1.5%. To reach an average false negative rate of 0%, we recommend *I*_1_ autocorrelation-based estimators (*E*_1_, *E*_2_, *E*_4_, and *E*_7_), where the price to be paid is an error of estimation of 0.8 bpm far from the expected accuracy of 0.25 bpm.

### 3.2. Results Obtained on Real Signals

We recorded 580 minutes for the analysis of real Doppler signals. We selected areas where signals had the cardiac activity signature. The performance levels on these signals were evaluated on the envelopes of both the nondirectional and the directional signals. The FHR estimation obtained with a commercial device (Oxford Sonicaid) was used as a reference to evaluate sensitivity which was evaluated for each estimator. All estimators were evaluated using a size of *W* = 4096 ms.

The results obtained for all the signals are presented in Table 3. The estimators based on directional signals (*E*_1_, *E*_4_, *E*_3_, and *E*_6_) provided a higher level of sensitivity compared to those which used nondirectional signals (*E*_7_, *E*_8_). In the case of directional signals, the results of *I*_1_ and *I*_2_ were close but slightly better for *I*_1_. This result confirmed the results obtained by simulations. We therefore recommend the use of estimators based on *I*_1_ calculated on the directional signals (*E*_1_, *E*_4_).

Sensitivity was improved using the combination method. The sensitivity of estimator *E*_2_ was 95.5% (see Table 3). Using the combination method, the sensitivity increased to about (95.5%–88.5%) ≈ 7% compared to directional signals *E*_4_ and to about (95.5%–81.8%) ≈ 14% compared to nondirectional signals *E*_7_.

## 4. Discussion and Conclusion

In this study, we focused on different settings of the estimators (window size, lag) that ensured a fetal heart rate estimation with a maximum authorized error of 0.25 bpm. We found in simulation that only estimators based on *I*_1_ and directional signals could ensure such an accuracy of 0.25 bpm. The size necessary in this case was *W* = 4096 ms.

Note that, although we proposed synthetic signals that were as realistic as possible, we are aware that the plotted performance levels are representative only of our simulations and not of all cases encountered in practice. It is likely that the performance levels of the algorithms tested can be reduced in the presence of artefacts. However, the 95% sensitivity obtained from real signals suggests that our proposed estimators may be trusted.

In the case of real signals, the sensitivity was quantified. Since in our study the estimated SNR on the real signals was greater than the threshold of 6 dB (deduced on simulated signals) required to reach the desired accuracy of 0.25 bpm, a denoising filter was not necessary. However, in cases of a SNR lower than 6 dB, a denoising process (Wiener, wavelet) could be introduced.

Sensitivity was quantified using a *W* size of 4096 ms. We found that the estimators *E*_1_, *E*_2_, and *E*_4_ based on *I*_1_ had slightly greater sensitivity than those based on *I*_2_. We therefore recommend the use of *I*_1_.

Various cases were considered on the basis of this study, that is, those that do not require a precise estimate of the fetal heart rate and those for which accuracy is critical. The accuracy of fetal heart rate estimation in the first case is not important but the false negative rate should be as low as possible. For example, if the goal of a monitoring system is simply to verify that the fetal heart rate is in the normal range (110–160 bpm), very high accuracy is not needed. In this case, an error of estimation of 0.8 bpm is sufficient. Our computations showed that for an error of estimation of 0.8 bpm, a zero false negative rate in the zones when the rhythm is quasi-constant could be ensured. In the second case, an error of estimation of 0.25 bpm is required for a system in which the goal is not only to estimate the heart rate, but also to evaluate fetal wellbeing. Our study showed that for this type of system, the false negative rate may be slightly higher than zero. It is important to note that this error of estimation was guaranteed for a quasi-constant heart rate. This is not a constraint for such a system, since the variability of fetal heart rate must be evaluated in these ranges to predict fetal distress.

Applied to real signals, the estimators based on *I*_1_ provided sensitivity close to those of *I*_2_, and the most efficient of these estimators were those that used directional signals (*E*_1_, *E*_2_, and *E*_4_).

A 7% increase in sensitivity compared to estimators based on individual directional signals was possible when we combined the two heart rates calculated on the directional signals. A 14% increase in sensitivity compared to estimators based on individual nondirectional signals was possible when we combined the two heart rates calculated on the directional signals. When a combination was used, both signals were processed in parallel, thus doubling the number of operations.

The good levels of performance of our estimator based on this combination suggest first that it can be adapted to multitransducer, multichannel configurations and second that such an estimator will improve fetal diagnosis.

## Figures and Tables

**Table 1:** Statistics evaluated using (2143) patterns: *M*_*i*_ represents the statistics of the maxima, where *i* = 1,…, 4,  *dP*_1_ *P*_2_, *dP*_2_ *P*_3_, *dP*_3_ *P*_4_ represent the differences between the positions of two adjacent maxima over time; *dM*_2_ *M*_1_, *dM*_1_ *M*_4_, *dM*_4_ *M*_3_ represent statistical differences between two adjacent maxima over time, and *T* represents the statistics of the peak durations. We found that the statistics of the four peaks of *T*_*i*_, *i* = 1,…, 4, were identical.

	Law	
	Gaussian	Uniform
*M*_1_	89.06 ± 31.48	
*M*_2_	69.70 ± 21.84	
*M*_3_	54.80 ± 19.21	
*M*_4_	36.28 ± 18.28	
*dM*_2_ *M*_1_		(5–50)
*dM*_1_ *M*_3_		(5–50)
*dM*_3_ *M*_4_		(5–40)
*dP*_1_ *P*_2_	41.50 ± 18.18	
*dP*_2_ *P*_3_	92.92 ± 27.76	
*dP*_3_ *P*_4_	47.81 ± 30.09	
*T* (ms)		(25–45)

**Table 2:** FHR error of estimation (bpm) obtained by all estimators tested for different configurations of SNR, false negative rate (FNR), and *W* = 4096 ms.

Estimators	FHR error of estimation (bpm)		
	*E*_1_, *E*_2_, *E*_4_, *E*_7_	*E*_3_, *E*_5_, *E*_6_	*E*_8_
FNR = 0%, 6 dB > SNR > 2 dB	0.8	4	6
FNR = 1.5%, SNR > 6 dB	0.25	—	—

**Table 3:** Sensitivity (%) of estimators for *W* = 4096 ms. *I*_1_ and *I*_2_ are the two autocorrelation estimators, respectively. *x*_*B*_, *x*_*F*_ are the envelopes of directional signals, and *x*_*e*_ is the envelope of the nondirectional signal. *Fus* indicates the combined estimator.

Sensitivity (*S*)	Estimators	
	*I*_1_	*I*_2_
*S* (*x*_*B*_)	88.50% (*E*_4_)	88.43% (*E*_6_)
*S* (*x*_*F*_)	86.63% (*E*_1_)	84.79% (*E*_3_)
*S* (*x*_*e*_)	81.79% (*E*_7_)	75.05% (*E*_8_)
*S* (*Fus*)	95.48% (*E*_2_)	94.93% (*E*_5_)
